# Towards a complex systems model of evidence for public health

**DOI:** 10.1136/bmjgh-2025-021061

**Published:** 2025-10-23

**Authors:** Karien Stronks, Morten Hulvej Rod, Harry Rutter, Naja Hulvej Rod

**Affiliations:** 1 Department of Public and Occupational Health, Amsterdam UMC/University of Amsterdam, Amsterdam, The Netherlands; 2 National Institute of Public Health, University of Southern Denmark, Copenhagen, Denmark; 3 Centre for 21st Century Public Health, University of Bath, Bath, UK; 4 Department of Public Health, University of Copenhagen, Copenhagen, Denmark

**Keywords:** public health, global health, health systems, prevention strategies

## Abstract

There has been growing interest in the adoption of complex systems approaches to tackle major public health challenges such as obesity. This perspective redirects focus from individually oriented and narrowly focused interventions towards strategies that reshape structural drivers of health and disease across multiple levels, offering novel avenues for enhancing population health and tackling health inequalities. Despite growing consensus on the use of a complex systems model of evidence to support these kinds of approaches, there remains limited agreement on the types of evidence required both to understand and to tackle complex public health problems. In this paper, we propose a complex systems model of evidence that combines three types of evidence—causal, intervention, implementation—across three dimensions of complex systems—mechanisms, dynamics and patterns. This model thus identifies nine categories of evidence: *causal evidence*, which explains the mechanisms behind public health problems, the dynamics driving changes therein and emerging health patterns; *intervention evidence,* which focuses on the set of actions that can modify these mechanisms and dynamics, including their intended and unintended consequences on health outcomes and *implementation evidence,* which addresses what is needed to implement systems change effectively, how systems adapt to these changes and how systems changes contribute to changes in health patterns. This complex systems model of evidence may serve as a guide to researchers and decision-makers when designing research programmes and evidence-based policies in response to complex public health problems.

**Summary box**

- In recent years, there has been growing consensus on the need for the adoption of complex systems approaches to tackle major public health challenges such as obesity, mental health and health inequality.
- There is still limited agreement on the types of evidence required both to understand and to tackle complex public health problems.
- We respond to this call by proposing a complex systems model of evidence that combines three types of evidence—causal, intervention, implementation—across three dimensions of complex systems—mechanisms, dynamics and patterns.
- This model can help researchers and decision-makers frame appropriate research questions when designing research programmes and developing evidence-based policies for complex public health problems.
- As a practical tool, it can serve as a framework for generating and synthesising meaningful, generalisable evidence to address complex public health problems, enabling a shift from evidence on individually oriented and narrowly focused interventions towards evidence on strategies that reshape structural drivers of health and disease across multiple levels.

## Introduction

 Scientific evidence plays an important role in enhancing population health, but current approaches to the generation and application of evidence are often insufficient to capture the complexity of contemporary public health challenges. The global failure to reduce health inequalities and the rising burden of chronic diseases are prompting a re-evaluation of the model of evidence underlying public health action.

In recent years, there has been growing interest in the adoption of complex systems approaches to address major societal challenges such as obesity.^1^,^5^ Within this paradigm, health is conceptualised as the outcome of a system of multiple exposures acting and interacting across multiple levels, from cell to society,^26^,^8^ emphasising ‘the importance of relationships and adaptive interactions of parts in the emergence of the whole’.^9^ This perspective redirects focus from individually oriented and narrowly focused interventions towards strategies that reshape structural drivers of health and disease across multiple levels, offering novel avenues for enhancing population health and tackling health inequalities.^2 6^

Despite growing consensus on the need for a complex systems model of evidence to support such approaches, there remains limited agreement on what *types of evidence* are required both to understand and to address complex public health problems. For example, evidence on single interventions developed in isolation has often proven ineffective when applied to complex public health problems.^2 6 8^ This raises critical questions: what alternative or additional forms of evidence are needed to build effective strategies? If, for example, the context in which a problem occurs determines which responses are likely to succeed, what kinds of evidence can support strategies that are adaptable across diverse contexts?

As a general model of evidence for public health, Brownson *et al*^10^ have argued that three types of evidence are needed:

- Type 1 (causal) evidence: on the effects of exposures on health outcomes.
- Type 2 (intervention) evidence: on the efficacy/effectiveness of interventions targeting these exposures.
- Type 3 (implementation) evidence: on the features of effective implementation of an intervention with proven effectiveness.

We believe that this typology, while helpful, would benefit from adaptations to address the complexity of contemporary problems. In this comment, we propose further development of Brownson’s typology towards the creation of a complex systems model of evidence. To this end, we draw on the Health Complexity Framework, which highlights the need to generate causal evidence on three core dimensions of complex problems—mechanisms, dynamics and patterns—to better *understand* the complex systems underlying a particular public health problem.^11^ *Mechanisms* refer to the interconnected causes of public health problems across multiple scales, from the cellular level to societal structures. *Dynamics* describe the ongoing processes—such as feedback and adaptation—that drive the evolution of these mechanisms over time. *Patterns* of health reflect the public health problems that emerge from underlying mechanisms and dynamics and cannot be predicted by examining individual components in isolation.^11^

In this paper, we extend this model of causal evidence to encompass intervention and implementation evidence. Specifically, we propose a model of evidence that integrates Brownson’s three *types* of evidence with the Health Complexity Framework’s three *dimensions* of complex systems. This results in a total of nine categories of evidence, presented as overarching research questions in table 1 and illustrated in the table and text with examples related to smoking. Table 2 provides further explanation and empirical illustrations from published research for all nine categories. This approach results in a complex system model of evidence that can support researchers and stakeholders involved in promoting public health to frame appropriate research questions.

**Table 1:** Complex systems model of evidence: conceptualisation and framing of overarching research questions

	Type 1	Type 2	Type 3
	Understanding complex public health problems	Identifying actions needed to modify these complex systems	Implementing systems changes
*Conceptualisation*			
Object of evidence	Public health problems and the complex system of mechanisms and dynamics underlying these.	Actions to modify complex systems that shape the public health problem(s) of interest.	Implementation of changes in complex systems that shape the public health problem(s) of interest.
Conceptualisation of core concepts	*Public health problems* are conceptualised as health patterns that emerge from mechanisms interacting across multiple scales combined with the dynamics that make them change over time.	A *set of actions* is needed to collectively bring about system change. Actions are conceptualised as ‘events in the system’.	The effectiveness of these actions in changing complex systems depends on the *context* as well as the process of *implementation*. The elements of actions-context-implementation are deeply interconnected.
*Framing of overarching research questions—and examples related to smoking*			
Mechanisms	Which mechanisms generate public health problems? *Example* Which mechanisms across individual, social and societal levels generate population patterns of smoking?	Which set of actions can modify the mechanisms that make up a complex system? *Example* Which set of actions can change the underlying mechanisms that shape population patterns of smoking?	What are the requirements for effectively implementing system change? *Example* Which conditions are necessary for the effective implementation of changes in the mechanisms that shape population patterns of smoking in local contexts?
Dynamics	Which dynamics drive changes in public health problems? *Example* What drives changes in population patterns of smoking?	Which set of actions can modify the dynamics of a complex system? *Example* Which set of actions can change the dynamic drivers of population patterns of smoking?	How does the system adapt to the implemented changes? *Example* In what ways do the drivers of population patterns of smoking adapt to the implementation of smoking prevention strategies?
Patterns	What are the emergent health patterns? *Example* How do smoking patterns vary across time and space, and how do they cluster in specific subgroups?	What are the intended as well as unintended consequences of the actions on health outcomes? *Example* What intended and unintended outcomes arise from smoking prevention strategies?	What are the outcome patterns in response to system changes? *Example* How have smoking prevention measures introduced in a specific context contributed to changes in local smoking patterns?

**Table 2:** Further explanation and examples of evidence and related research questions within the complex systems model of evidence

	Type 1 Understanding complex public health problems	Type 2 Identifying type of actions needed to modify these complex systems	Type 3 Implementing systems changes
Mechanisms	*We need evidence on:* multiscale mechanisms that generate emergent health patterns. *Example*^37^ *Research question:* what mechanisms underlie an increase in obesity? *Obtained evidence on mechanisms:* obesity spread in social networks.	*We need evidence on:* set of actions to modify these mechanisms, defined in terms of their function, based on a theory of change. *Example*^38^ *Research question:* What actions might reduce the abundance of unhealthy food around schools? *Underlying mechanism addressed by the actions: *power imbalance between large global food companies and smaller local companies. *Obtained evidence on actions:* multiple activities (workshop, group model building, etc), aimed at fostering a sense of shared responsibility for the food environment of school children among relevant actors in supermarkets, are likely to reduce the abundance of food in supermarkets around schools.	*We need evidence on:* the requirements for effectively implementing the intended systems change, with actions-context-implementation acting in concert. *Example*^38^ *Research question:* How can one increase the attractiveness of a public space for outdoor active play, from the perspective of adolescents? *Obtained evidence on implementation of system change:* co-creating a public outdoor space (=set of actions) to make it more appealing for outdoor active play, particularly from the perspective of adolescents, requires the active involvement of local civil servants (=context). These civil servants must be trained in co-creation techniques and provided with necessary support (=implementation process).
Dynamics	*We need evidence on*: the dynamics that drive changes in mechanisms and health patterns over time. *Example*^39^ *Research question:* What are the reinforcing feedback loops that underlie the relationship between exposure to adverse socioeconomic conditions and chronic stress? *Obtained evidence on dynamics:* socioeconomic disadvantage is linked with chronic stress through reinforcing feedback loops such as learnt helplessness.	*We need evidence on:* set of actions to modify these dynamics, defined in terms of their function, based on a dynamical theory of change. *Example*^18^ *Research question:* What actions are needed to improve living environments in deprived neighbourhoods? *Underlying dynamic addressed by the actions:* planting street trees might worsen the health of citizens if the trees are not well maintained as that might lead to an environment characterised by neglect and disrepair. *Obtained evidence on actions:* to improve inhabitants’ health, planting trees should be accompanied by ensuring adequate resources for their maintenance.	*We need evidence on:* how the complex system adapts as a consequence of the realised systems change. *Example*^19^ *Research question:* How does a system in which a sugar tax is implemented react to this implementation, and how does this reaction contribute to a weakening of the tax? *Obtained evidence on the implementation of system change:* a sugar tax policy led manufacturers in a commercial marketplace to adopt strategies to minimise its impact on product sales, which limits its potential impact to change dietary patterns.
Patterns	*We need evidence on:* emergent health patterns across time and space. *Example*^40^ *Research question:* Which trajectories of adverse childhood experience are related to increased mortality in early adult life? *Obtained evidence on patterns:* children with multiple adversities across social, health and family related dimensions have a markedly increased mortality risk in early adulthood.	*We need evidence on:* intended as well as unintended consequences of a set of actions on health. *Example*^41^ *Research question:* Do 30 km/h/20 mph speed limit measures improve population health? *Underlying mechanisms and dynamics addressed by the actions:* these measures might lead to (1) compensatory speeding elsewhere; (2) reduced driver attention (eg, using mobile phones) and (3) a false sense of safety among pedestrians that is not matched by drivers adhering to the speed limit. *Obtained evidence on actions*: 20 mph speed limits could lead to an increased rate of collisions and casualties, through the abovementioned mechanisms and dynamics.	*We need evidence on:* changes in health patterns in response to system changes and dynamics therein. *Example*^42^ *Research question:* How has the implementation of soft drinks industry levy (SDIL) in the UK shaped the trend in prevalence of obesity? *Obtained evidence on the implementation of system change:* implementation of the SDIL in the UK resulted in a decrease in obesity prevalence in girls aged 10–11 years, but less so in boys. Additional measures might be necessary for the latter to achieve the same impact.

### Type 1 evidence: understanding the complex system

In Brownson’s typology, type 1 evidence is concerned with establishing the causality underlying public health problems. Traditionally, public health research has aimed to establish singular causes, for example, by examining how behavioural risk factors, such as smoking, lead to diseases, such as lung cancer.^10^ While this approach has provided valuable knowledge, it often cannot stand alone for dealing with complex public health problems.^2^ The Health Complexity Framework provides a broader conceptualisation of the research questions that need to be addressed to understand complex public health problems, according to the abovementioned dimensions of complex systems (table 1).^11^

#### Mechanisms: Which mechanisms generate public health problems?

Evidence on *mechanisms* systematically describes the interconnectedness between relevant causes across multiple scales. Thus, we need to understand causality as something that is generated as an interplay of mechanisms across molecular, individual, group and societal scales.^12^ For example, the mechanisms underlying smoking as a public health problem operate both within the body and through the influence of psychosocial and commercial factors.

#### Dynamics: Which dynamics drive changes in public health problems?

To capture the ongoing changes of a complex system, there is a need for evidence on the *dynamics* that cause mechanisms to evolve over time through feedback and adaptation.^5 11^ For example, smoking behaviour may be reinforced by feedback loops between social exclusion and social norms, leading to higher smoking rates in certain populations.

#### Patterns: What are the emergent health patterns?

Evidence on the changing *patterns* of health that result from the underlying mechanisms and their evolution over time captures the emergence of public health problems that cannot be predicted from individual elements of the underlying system.^5 11^ An example of this kind of evidence is that smoking has been found to cluster with other types of risk behaviour.

### Type 2 evidence: identifying *actions* that can modify the complex system

Modifying the emergent patterns of a complex health problem towards a more desirable state, such as reduced health inequality, requires reshaping the mechanisms and dynamics that make up the complex system underlying public health problems. In the traditional model of evidence for public health, type 2 evidence relates to the efficacy or effectiveness of distinct and fixed interventions targeting specific risk factors such as ‘price increases on cigarettes reduce smoking rates’.^10^ But this approach may be insufficient if it, for example, fails to take account of system adaptations, such as increased marketing efforts by the tobacco industry. Thus, instead of these isolated interventions, there is a need for a *set of actions* to collectively bring about such systems change.^13^ Actions capable of this reshaping can be conceptualised as ‘events’ or ‘disruptions’ within the system they aim to change.^14^ Type 2 evidence indicates how certain actions are likely to modify the underlying *mechanisms* and *dynamics*, and how, subsequently, effects on health *patterns* might be achieved (table 1).

#### Mechanisms: Which set of actions can modify the mechanisms that make up a complex system?

In order to identify a *set of actions* that can create change, we need to build on our understanding of the mechanisms underlying complex problems. Given the conceptualisation of actions as ‘events in a system’, we need evidence on how specific actions are likely to modify these mechanisms, and in turn modify the system. In order for this evidence to be generalisable, it is important to define these actions in terms of their *function* within the system—that is, the changes they produce in the underlying mechanisms—rather than focusing solely on their content or their direct effects on a simple causal outcome. This function is grounded in an intervention theory or theory of change.^15 16^ For example, in the context of preventing youth smoking, a recent review assessed the effectiveness of school-based interventions in terms of characteristics of the intervention, such as mode of delivery or the type of intervention provider.^17^ To extend this analysis towards a complex systems-based perspective, these interventions would need to be evaluated in terms of their function within the system—such as their ability to create and sustain social norms that discourage smoking among young people.

#### Dynamics: Which set of actions can modify the dynamics of the system?

When identifying actions, evidence on how they modify the *system dynamics* underpinning specific health problems is also required, again, defined in terms of the function of the actions.^18^ An example of this type of evidence might be a finding that ‘rebellious smoking’ among young people—smoking in friendship groups outside school to express toughness—is a common dynamic, and punishing this behaviour reinforces it.

#### Patterns: What are the intended as well as unintended consequences of the actions on health outcomes?

In complex systems, the relation between intended modifications in the mechanisms and dynamics that result from specific actions on the one hand, and desired changes in health patterns on the other, may not be self-evident. Due to the core features of complex systems of interconnectedness and adaptation, an intervention in a complex system may have *unintended* consequences alongside the hoped-for outcomes.^19^ As a consequence, actions that have the intention to improve health might actually achieve the opposite. Monitoring the intended as well as unintended consequences is essential to achieving improved overall health outcomes.^20^ An example of this type of evidence might be a finding that strategies to change parental behaviour have protected children from secondhand smoking, but have also contributed to increased stigmatisation of mothers who continue to smoke, thereby worsening their mental health.

### Type 3 evidence: implementing systems change

In the traditional model of public health evidence, implementation evidence focuses on the key features of effectively implementing a specific intervention with proven effectiveness.^10^ A typical example would be: ‘effective implementation of tobacco sales regulations requires enforcement by officers with the authority to impose sanctions on retailers who fail to comply with the law’. However, addressing complex public health problems requires evidence that goes beyond understanding what is needed to implement specific actions; it also involves understanding what is necessary for *system-level change* to occur.^21^ In other words, it specifies how the intended system change can be effectively implemented (table 1).^13^ More specifically, type 3 specifies the requirements for changing the mechanisms that constitute the system, how this change impacts on the dynamics of the system and how, subsequently, changes in health patterns occur. When intended system changes involve fundamental shifts in the system’s function or purpose, the term systems *transformation* is typically used.^6^ Here, we use system change to encompass both modifications to specific elements (while maintaining the core structure) and more fundamental, transformative changes.^22^ Building on the example above, this would demonstrate the need for evidence for *how* to create a system in which there are sufficient numbers of well-resourced enforcement officers with the authority to impose robust and effective sanctions on non-compliant retailers.

#### Mechanisms: What are the requirements for effectively implementing systems change?

In driving systems change, we build on type 2 evidence regarding the set of actions that can modify the mechanisms of the system. The effectiveness of these actions in bringing about actual change depends on both the *context* in which they are implemented and the *implementation process*.^23^ Context includes factors such as societal norms, beliefs and values within the population at stake, and among the people working in the organisations that carry out the actions.^16 24^ The process of implementation refers to the endeavours made to implement the actions within a specific setting.^21 25^

When implementing system change, actions, context and the implementation process are deeply interconnected and act in concert to impact on mechanisms.^14 23^ In fact, the construct of actions-context-implementation can itself be viewed as a complex system, with independent elements influencing one another.^26^ To implement systems change effectively, evidence is required on how different configurations of these elements might promote systems change. An example of this kind of evidence would be a finding that political priority providing financial opportunities, staff structure and time resources in a specific setting is essential for the implementation of a smoking ban.

#### Dynamics: How does the system adapt to the implemented changes?

Actions intended to create change within systems are more likely to achieve their intended effects if they are able to adapt dynamically in response to changes that result within the system.^9^ For example, if stakeholders in the targeted system have vested interests that drive resistance to systems change, it will be important to identify evidence on approaches to counter this resistance. This type of evidence on how a system evolves in response to interventions can be grounded in a *dynamic* theory of change that considers ‘intervention and system coevolution over time’.^18 20^ An example of this evidence might be a finding that the introduction of new, unregulated nicotine-containing products by the tobacco industry—allowing them to maintain consumer interest while bypassing stricter regulations on traditional tobacco products—needs to be met by the government updating tobacco control laws to include all nicotine products.

#### Patterns: What are the outcome patterns in response to system changes?

The final piece of evidence shows whether changes in the system and dynamics therein are actually followed by changes in health patterns. Given the interconnectedness between actions-context-implementation, it is impossible to quantitatively *attribute* an observed change in that health problem to specific actions. Instead, the highest attainable goal is to clarify how the actions and the way they have been implemented in a specific context may have *contributed* to the change.^27^ In other words, how reasonable is it that the set of actions contributed to the observed system changes as well as the emerging patterns in health? If that appears not to be the case, the theory of change should be adapted accordingly, as a basis for a novel set of actions.^20^ In the smoking example, this might be evidence that the denormalisation of smoking, itself driven by a comprehensive set of antismoking measures, has contributed to a decline in smoking-related diseases.

### Conclusions and next steps

We propose a complex systems model of evidence that combines three types of evidence with the three dimensions of complex systems as outlined in the Health Complexity Framework. This integration yields nine distinct types of evidence: *causal evidence* explains the mechanisms behind public health problems, the dynamics driving changes therein and emerging health patterns; *intervention evidence* focuses on the set of actions that can modify these mechanisms and dynamics, including their intended and unintended consequences on health outcomes and *implementation evidence* addresses what is needed to implement systems change effectively, how systems adapt to these changes and on how system changes contribute to changes in health patterns.

These nine types of evidence can serve as a model for systematically generating and synthesising meaningful, generalisable evidence across diverse studies and actions aimed at understanding and addressing complex public health problems. More specifically, this model can help researchers, decision-makers and other stakeholders frame appropriate research questions when designing research programmes and developing evidence-based policies for complex health problems. For example, if the goal is to generate or synthesise evidence on actions to prevent smoking, the model highlights the importance of understanding the mechanisms and dynamics that drive smoking trends, as well as identifying actions capable of modifying these mechanisms and dynamics. This approach stands in contrast to the traditional model of evidence, which focuses primarily on identifying risk factors for smoking and assessing the effectiveness of specific interventions targeting those factors.

This model builds on the concept of ‘building a dry stone wall’ introduced by Ogilvie *et al* to make sense of evidence in public health research. Ogilvie *et al* advocate for viewing evidence as ‘pluralist mosaics whose strength derives from the complementary of their components’.^28^ The complex systems model of evidence extends this idea by specifying the *types of evidence* that constitute these components, for causal, intervention and implementation evidence. Systematically addressing each type of evidence helps avoid blind spots in research and policy. Consistent with the ‘dry stone wall’ metaphor, the overarching research questions within each evidence type are likely to span multiple aspects. For example, evidence on mechanisms underlying smoking may encompass individual-level factors, such as stress, as well as macro-level determinants such as commercial influences.

We have presented the types of evidence in a logical sequence. For example, we argue that understanding system mechanisms forms the foundation for identifying actions to modify these mechanisms, which then leads to developing a strategy to implement system change in a specific context. In practice, however, these types of evidence might inform each other in various orders. For example, evidence on how actions impact mechanisms (intervention evidence) can provide novel insights that reshape the need for specific causal evidence. In fact, addressing public health problems often requires an iterative approach, moving back and forth between causal evidence, evidence on actions and evidence on the implementation of system change.

The complex systems model of evidence is informed by empirical examples that illustrate its practical relevance. Table 2 presents a selection of such examples, demonstrating how the model can be applied across different contexts and highlighting the diverse ways in which it can guide the generation and synthesis of evidence. This overview also highlights how some types of evidence are far more studied than others. Evidence on the implementation of system changes seems to be particularly limited. For example, a scoping review of reviews on whole system approaches to healthy weight^29^ found that the available evidence on the implementation of system change predominantly consists of a list of contextual factors that promote implementation. Such lists clearly fail to capture the inseparability of actions, context and implementation process, as emphasised in the proposed complex systems model of evidence. Systematically assessing the outlined nine types of evidence can hopefully prevent such blind angles in the future.

Having outlined the need for a new model of evidence, the next question is: How can we promote the production of rigorous, transparent and systematic evidence for each of the nine types of evidence, ensuring they meet the highest scientific standards? Addressing this requires a pluralistic (mosaic) approach to causality and methodological pluralism according to which there is no pre-determined hierarchy of methods and where different methods can help us understand problems from different angles.^30 31^ This empirical evidence needs to be supplemented with theoretical evidence, such as theoretical studies that articulate the theory of change underlying specific actions.^32 33^

Uncertainty is an inherent part of a complex systems model of evidence due to the interconnectedness and dynamism of complex phenomena. Rather than aiming for an unachievable objective of certainty, we acknowledge that navigating uncertainty is integral to effectively addressing public health problems. This requires engaging with disparate types of evidence, derived from a wide range of methodologies. In line with the previously introduced ‘dry stone wall’ metaphor,^28^ this process involves assembling pieces of evidence into a stable structure that can adapt to uneven ground—unlike a regular brick wall where uniform bricks fit neatly together but are less suited to uneven terrain, such as the ‘messiness’ of public health.

We advocate for the inclusion of multiple stakeholder perspectives across all types of evidence.^33^ Embracing a multistakeholder approach helps ensure that research objectives and questions remain closely aligned with real-world relevance.^34^ Stakeholders should not be viewed merely as recipients of evidence, but instead as active participants in the research process—and, in many cases, as integral members of research teams.^35 36^

To conclude, by proposing a complex systems model of evidence for public health, this paper contributes to the advancement of systematic evidence generation within public health, with the ultimate aim to inform and guide stakeholders involved in promoting public health. Next steps involve developing the methodological standards for generating scientific evidence for each of the nine pieces of evidence. Embracing the proposed complex systems model of evidence in combination with a multistakeholder approach ensures that our research objectives and questions remain relevant to challenges in public health practice.

## Data Availability

There are no data in this work.

## References

[1] UK Government’s Foresight Programme (2007). Tackling obesities: future choices – project report.

[2] Rutter H, Savona N, Glonti K (2017). The need for a complex systems model of evidence for public health. The Lancet.

[3] Swinburn B (2019). Power Dynamics in 21st-Century Food Systems. Nutrients.

[4] Stronks K, Nicolaou M (2018). Embracing complexity in social epidemiology. Lancet Public Health.

[5] Rickles D, Hawe P, Shiell A (2007). A simple guide to chaos and complexity. J Epidemiol Community Health.

[6] Gadsby EW, Wilding H (2024). Systems thinking in, and for, public health: a call for a broader path. Health Promot Int.

[7] Stronks K, Crielaard L, Rod NH, Ahrens W, Pigeot I (2024). Handbook of epidemiology.

[8] Rusoja E, Haynie D, Sievers J (2018). Thinking about complexity in health: A systematic review of the key systems thinking and complexity ideas in health. J Eval Clin Pract.

[9] Greenwood-Lee J, Hawe P, Nettel-Aguirre A (2016). Complex intervention modelling should capture the dynamics of adaptation. BMC Med Res Methodol.

[10] Brownson RC, Fielding JE, Maylahn CM (2009). Evidence-based public health: a fundamental concept for public health practice. Annu Rev Public Health.

[11] Rod NH, Broadbent A, Rod MH (2023). Complexity in Epidemiology and Public Health. Addressing Complex Health Problems Through a Mix of Epidemiologic Methods and Data. Epidemiology (Sunnyvale).

[12] Uleman JF, Stronks K, Rutter H (2024). Mapping complex public health problems with causal loop diagrams. Int J Epidemiol.

[13] Pescud M, Rychetnik L, Allender S (2021). From Understanding to Impactful Action: Systems Thinking for Systems Change in Chronic Disease Prevention Research. *Systems*.

[14] Hawe P, Shiell A, Riley T (2009). Theorising Interventions as Events in Systems. *American J of Comm Psychol*.

[15] Hawe P (2015). Lessons from complex interventions to improve health. Annu Rev Public Health.

[16] Minary L, Alla F, Cambon L (2018). Addressing complexity in population health intervention research: the context/intervention interface. J Epidemiol Community Health.

[17] Hossain S, Tattan-Birch H, Beard E (2025). Evaluating School-Based Interventions for Preventing and Reducing Tobacco Use Among Adolescents in Low- and Middle-Income Countries: A Systematic Review and Meta-Analysis. Am J Prev Med.

[18] Alvarado M, Garrett J, Fullam J (2024). Using causal loop diagrams to develop evaluative research propositions: opportunities and challenges in applications to nature‐based solutions. Syst Dyn Rev.

[19] Alvarado M, Marten R, Garcia L (2023). Using systems thinking to generate novel research questions for the evaluation of sugar-sweetened beverage taxation policies. BMJ Glob Health.

[20] Luna Pinzon A, Stronks K, Dijkstra C (2022). The ENCOMPASS framework: a practical guide for the evaluation of public health programmes in complex adaptive systems. Int J Behav Nutr Phys Act.

[21] Braithwaite J, Churruca K, Long JC (2018). When complexity science meets implementation science: a theoretical and empirical analysis of systems change. BMC Med.

[22] Crielaard L, Nicolaou M, Brown AD (2025). Systems approaches in public health: beyond mapping the causes. Int J Behav Nutr Phys Act.

[23] Pfadenhauer LM, Gerhardus A, Mozygemba K (2017). Making sense of complexity in context and implementation: the Context and Implementation of Complex Interventions (CICI) framework. *Implementation Sci*.

[24] Rodgers M, South E, Harden M (2025). Contextual factors in systematic reviews: understanding public health interventions in low socioeconomic status and disadvantaged populations. Arch Public Health.

[25] Whelan J, Fraser P, Bolton KA (2023). Combining systems thinking approaches and implementation science constructs within community-based prevention: a systematic review. Health Res Policy Syst.

[26] Koorts H, Rutter H (2021). A systems approach to scale-up for population health improvement. *Health Res Policy Sys*.

[27] Ling T (2012). Evaluating complex and unfolding interventions in real time. Evaluation (Lond).

[28] Ogilvie D, Bauman A, Foley L (2020). Making sense of the evidence in population health intervention research: building a dry stone wall. BMJ Glob Health.

[29] Breslin G, Fakoya O, Wills W (2024). Whole systems approaches to diet and healthy weight: A scoping review of reviews. PLoS One.

[30] Russo F, Broadbent A, Castellani B, Illari P, Russo F (2025). The Routledge handbook of causality and causal methods.

[31] Haynes A, Rychetnik L, Finegood D (2020). Applying systems thinking to knowledge mobilisation in public health. Health Res Policy Sys.

[32] McGill E, Er V, Penney T (2021). Evaluation of public health interventions from a complex systems perspective: A research methods review. Soc Sci Med.

[33] Astbury CC, Lee KM, McGill E (2023). Systems Thinking and Complexity Science Methods and the Policy Process in Non-communicable Disease Prevention: A Systematic Scoping Review. Int J Health Policy Manag.

[34] Nguyen L-K-N, Kumar C, Jiang B (2023). Implementation of Systems Thinking in Public Policy: A Systematic Review. *Systems*.

[35] Luna Pinzon A, Stronks K, Verhoeff A (2025). Applying a participatory system dynamics approach to childhood overweight and obesity in the local context: reflections from the LIKE project. *Health Res Policy Sys*.

[36] Reed JE, Green S, Howe C (2019). Translating evidence in complex systems: a comparative review of implementation and improvement frameworks. Int J Qual Health Care.

[37] Christakis NA, Fowler JH (2007). The spread of obesity in a large social network over 32 years. N Engl J Med.

[38] Luna Pinzon A, Waterlander W, de Pooter N (2024). Development of an action programme tackling obesity-related behaviours in adolescents: a participatory system dynamics approach. *Health Res Policy Sys*.

[39] Crielaard L, Nicolaou M, Sawyer A (2021). Understanding the impact of exposure to adverse socioeconomic conditions on chronic stress from a complexity science perspective. BMC Med.

[40] Rod NH, Bengtsson J, Budtz-Jørgensen E (2020). Trajectories of childhood adversity and mortality in early adulthood: a population-based cohort study. Lancet.

[41] Cleland CL, Baker G, Turner K (2021). A qualitative exploration of the mechanisms, pathways and public health outcomes of a city centre 20mph speed limit intervention: The case of Belfast, United Kingdom. Health Place.

[42] Rogers NT, Cummins S, Forde H (2023). Associations between trajectories of obesity prevalence in English primary school children and the UK soft drinks industry levy: An interrupted time series analysis of surveillance data. PLoS Med.

